# *Arabidopsis*‐expressing lysine‐null SUMO1 reveals a non‐essential role for secondary SUMO modifications in plants

**DOI:** 10.1002/pld3.506

**Published:** 2023-07-16

**Authors:** Thérèse C. Rytz, Juanjuan Feng, Jessica A. S. Barros, Richard D. Vierstra

**Affiliations:** ^1^ Department of Biology Washington University in St. Louis St. Louis Missouri USA; ^2^ Benson Hill Inc. St. Louis Missouri USA; ^3^ State Key Laboratory of Cotton Biology, School of Life Sciences Henan University Kaifeng China

**Keywords:** *Arabidopsis*, heat stress, mass spectrometry, SUMO, SUMOylation, ubiquitin

## Abstract

The reversible conjugation of small ubiquitin‐like modifier (SUMO) to other proteins has pervasive roles in various aspects of plant development and stress defense through its selective attachment to numerous intracellular substrates. An intriguing aspect of SUMO is that it can be further modified by SUMOylation and ubiquitylation, which isopeptide‐link either or both polypeptides to internal lysines within previously bound SUMOs. Although detectable by mass spectrometry, the functions of these secondary modifications remain obscure. Here, we generated transgenic *Arabidopsis* that replaced the two related and essential SUMO isoforms (SUMO1 and SUMO2) with a lysine‐null SUMO1 variant (K0) immune to further SUMOylation/ubiquitylation at these residues. Remarkably, homozygous *SUMO1(K0) sumo1 sumo2* plants developed normally, were not hypersensitive to heat stress, and have nearly unaltered SUMOylation profiles during heat shock. However, subtle changes in tolerance to salt, paraquat, and the DNA‐damaging agents bleomycin and methane methylsulfonate were evident, as were increased sensitivities to ABA and the gibberellic acid biosynthesis inhibitor paclobutrazol, suggesting roles for these secondary modifications in stress defense, DNA repair, and hormone signaling. We also generated viable *sumo1 sumo2* lines expressing a SUMO1(K0) variant specifically designed to help isolate SUMO conjugates and map SUMOylation sites, thus offering a new tool for investigating SUMO *in planta*.

## 1 INTRODUCTION

Plants employ a wide array of post‐translational modifiers to regulate the location, functionality, interactions, and/or half‐lives of their resident proteins. Particularly influential is the ~100‐amino‐acid protein small ubiquitin‐like modifier (SUMO), a structural relative of ubiquitin (Ub) that likewise becomes dynamically conjugated to a plethora of intracellular proteins (Augustine & Vierstra, 2018; Benlloch & Lois, 2018; Geiss‐Friedlander & Melchior, 2007). Once bound, the SUMO moiety can modify the behavior of its substrates often in substrate‐specific manners, including altering their enzymatic activity, changing their cellular location, promoting or inhibiting association with other proteins, and/or stimulating or blocking subsequent modification by Ub. In many cases, the appended SUMO regulates protein–protein interactions through its binding to other proteins harboring SUMO‐interacting Motifs (SIMs) (Yau et al., 2021). Ultimately, numerous cellular events are impacted, many of which are important to genome organization, transcription, RNA splicing and metabolism, signal transduction, and plant stress defense (Augustine & Vierstra, 2018; Benlloch & Lois, 2018; Morrell & Sadanandom, 2019).

SUMO addition occurs through an ATP‐dependent conjugation cascade sequentially involving the heterodimeric SAE1/SAE2 SUMO‐activating enzyme (E1), the SCE1 SUMO‐conjugating enzyme (E3), and a small set of SUMO‐ligases (E3s) that connect the charged, thiolester‐linked E2‐SUMO adduct with specific substrates and then facilitate SUMO transfer onto accessible lysines (Geiss‐Friedlander & Melchior, 2007; Psakhye & Jentsch, 2012). The final product is a SUMO moiety covalently attached through an isopeptide bond between the C‐terminal glycine carboxyl group of SUMO and one or more ε‐amino lysl groups within the substrate. *Arabidopsis* encodes at least three E3 types—SIZ1, MMS21, and PIAL1 and 2, which ultimately help modify more than a thousand substrates as detected by mass spectrometry (MS) (Miller et al., 2010, 2013; Rytz et al., 2018). In some cases, SUMOylation can also occur on multiple proteins in close proximity to the substrate directly recognized by the E3 through a SUMO “spray” mechanism (Psakhye & Jentsch, 2012). The pathway also includes a family of deSUMOylating enzymes that can release both the substrate and SUMO moieties in free, functional forms by specific cleavage of the isopeptide bond, thus allowing for reversible modifications (Kunz et al., 2018; Morrell & Sadanandom, 2019).

Plants typically express several SUMO isoforms. Within the eight‐membered family in *Arabidopsis*, only four appear to be transcriptionally active (Kurepa et al., 2003; van den Burg et al., 2010). Included are the close paralogs—SUMO1 and SUMO2 (94% identical), which are best expressed, essential genetically, and responsible for most, if not all, of the SUMOylation seen in vivo especially under stress such as heat shock (Augustine et al., 2016; Kurepa et al., 2003; Saracco et al., 2007). SUMO3 and SUMO5 by contrast, are loosely constrained by sequence (only 48% and 35% identical to SUMO1) and expressed at low levels with little evidence of functionality besides a possible role for SUMO3 in salicylic acid‐mediated defense and/or antagonizing the actions of SUMO1/2 (Ingole et al., 2021; van den Burg et al., 2010). It still remains to be conclusively demonstrated that SUMO3 and SUMO5 actually become conjugated to other proteins *in planta*.

Like Ub, an intriguing feature of the bound SUMO is that it can be further modified by SUMOylation and/or ubiquitylation in which the first SUMO becomes modified at accessible lysines by additional SUMOs or Ubs (Keiten‐Schmitz et al., 2019; Vertegaal, 2010). Ultimately, it is possible that chains of SUMO or SUMO‐Ub are assembled with either linear or branched architectures using these lysine‐glycine isopeptide connections. In yeast and mammals, polymer concatenation mostly occurs at a flexible 20‐amino‐acid N‐terminal extension upstream of the β‐grasp Ub‐fold in SUMO, which is predicted to be intrinsically disordered (Figure S1a). Assembly of SUMO–SUMO chains is likely encouraged by SIZ1‐ and PIAL1/2‐type E3s that harbor binding sites for both the E2‐SUMO adduct and for free SUMO via SIMs (Jansen & Vertegaal, 2021; Keiten‐Schmitz et al., 2019). For SUMO‐Ub linkages, a family of SUMO‐targeted Ub ligases (StUbLs) directs Ub addition onto previously bound SUMOs, which includes human RNF4 and RNF11 and the yeast Slx5/8 heterodimer (Kumar et al., 2017). Assembly of the SUMO–SUMO polymers can be counterbalanced by a subfamily of deSUMOylating enzymes that includes Semp6 and Semp7 in humans and Ulp2 in yeast, thereby reversing this secondary modification (Kunz et al., 2018).

At present, the identities of the proteins modified with SUMO–SUMO and SUMO‐Ub polymers are mostly unclear as are the functions of the added SUMOs/Ubs (Keiten‐Schmitz et al., 2019; Vertegaal, 2010). Through the analysis of SUMO mutants that replaced all lysines with arginines and thus blocked subsequent additions, it appears that such secondary modifications are not essential in the yeast *Saccharomyces cerevisiae*, with the caveat that a deletion mutant of the Upl2 isopeptidase which cannot disassemble such polymers is phenotypically compromised and hyperaccumulates high‐molecular mass SUMO conjugates (Bylebyl et al., 2003). In *Schizosaccharomyces pombe*, lysine‐null SUMO strains are also viable but display nuclear defects and a hypersensitivity to genotoxic stress (Skilton et al., 2009). In mammalian cells, SUMO‐Ub chains have been implicated in various aspects of centromere organization, chromatin cohesion, DNA repair, and replication likely through the action of StUbLs (Jansen & Vertegaal, 2021; Keiten‐Schmitz et al., 2019). Whether such polymers are essential to mammals remains unclear but they preferentially assemble in cells under stress, suggesting protective functions (Vertegaal, 2010).

In plants, the actions of SUMO–SUMO and SUMO‐Ub concatemers are not yet understood. Miller et al. (2010, 2013) detected such species for SUMO1/2 by tandem mass spectrometric (MS/MS) analysis of SUMOylated proteins both before and after acute heat or oxidative stress. Their approach was to exploit a functional *Arabidopsis* SUMO1 variant where the C‐terminal HQTGG sequence was modified to include a trypsin cleavage site after residue 89, thus allowing detection by MS/MS of both SUMOylated SUMO and ubiquitylated SUMO through the identification of trypsinized peptides bearing footprints of substrate lysines isopeptide modified with either SUMO (QTGG; 326 m/z) or Ub (GG; 114 m/z) fragments. At least for the assembly of SUMO‐Ub adducts, a small gene family encoding possible orthologs of yeast and mammalian StUbLs have been detected in plant genomes (six in *Arabidopsis*); one could partially rescue the genome integrity defects seen in the *rfp1/rfp2* StUbL mutant from *S. pombe* (Benlloch & Lois, 2018; Elrouby, 2015; Elrouby et al., 2013), while another has been indirectly linked to histone methylation (Hale et al., 2016).

Here, we attempted to address the importance of SUMO–SUMO and SUMO‐Ub polymers through the creation of *Arabidopsis* SUMO1/2 lines potentially missing these secondary modifications. This was accomplished by rescuing mutants eliminating the pair with a *SUMO1* variant harboring arginine replacements for all seven lysines, which would block these additions but still retain functionality at least as seen in vitro (Augustine et al., 2016; Zhang et al., 2021). Remarkably, these *SUMO1(K0) sumo1–1 sumo2–1* plants were phenotypically normal when grown under optimal conditions and showed similar SUMOylation patterns before, during, and after heat shock. However, altered stress tolerance and hormone sensitivities were seen, suggesting that these secondary SUMO modifications do have more subtle roles. We also generated *Arabidopsis* lines expressing a 6His‐tagged SUMO1(K0) mutant bearing substitutions designed to help map SUMO1/2 conjugation sites (Miller et al., 2010, 2013). Preliminary studies revealed that this 6His‐(M1R)‐K0(H89R) variant also retained functionality *in planta*, thus providing a new tool to dissect SUMOylation by MS/MS approaches.

## 2 RESULTS

### 2.1 SUMO lysines are not essential in *Arabidopsis*

**FIGURE 1:**
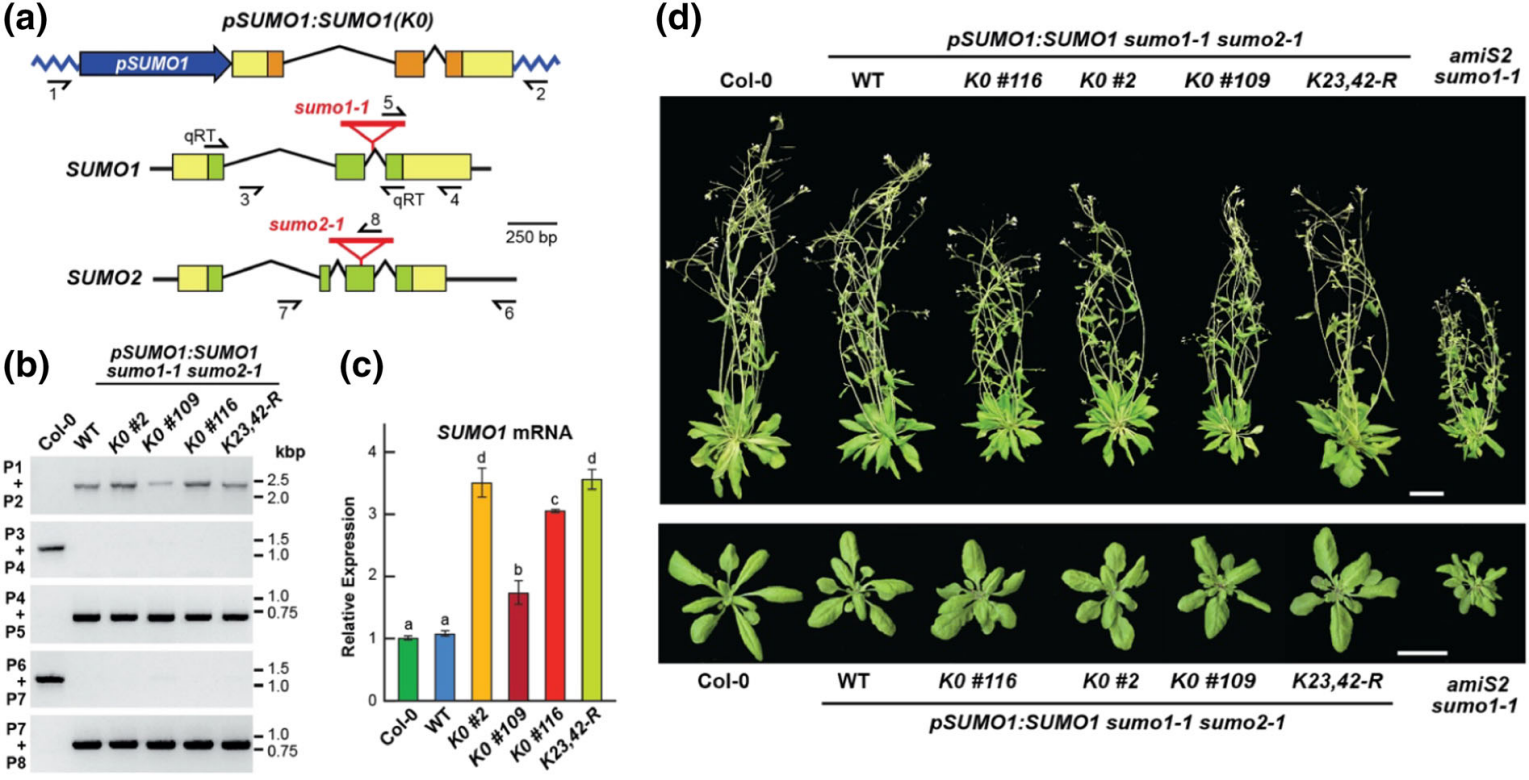
Lysine‐null SUMO1 variant rescues the *Arabidopsis* embryo‐lethal *sumo1 sumo2* mutant. (a) Diagram of the *pSUMO1:SUMO1(K0)* transgene expressing a lysine‐null variant of *Arabidopsis* SUMO1 in which all seven lysines were replaced with arginines (K0) (see Figure S1). (top) Organization of the *SUMO1(K0)* transgene. (bottom) Organization of the endogenous *SUMO1* and *SUMO2* loci showing the positions of the T‐DNA insertions (red triangles) that disrupt expression. The *SUMO1* promoter is shown in blue. Untranslated and coding regions are in yellow and orange boxes, respectively. Introns are shown by the broken lines. Half arrows locate the positions of the primers used for genotyping and qRT‐PCR (qRT) in panels (b) and (c), respectively. (b) Genotype confirmation of Col‐0 and the *SUMO1* transgenic lines by PCR. Genomic DNA was isolated from 27‐day‐old wild‐type *Arabidopsis* Col‐0, and independent *sumo1–1 sumo2–1* transgenic seedlings expressing wild‐type SUMO (WT), the SUMO1(K0) variant (*K0 #2*, *K0 #109*, and *K0 #116*), or the *K23,42‐R* variant harboring lysine→arginine substitutions at positions 23 and 42. (c) Relative transcript abundance for the *SUMO1* loci in the genotypes described in panel (b). Transcript levels were quantified by qRT‐PCR of total RNA extracted from 7‐day‐old seedlings, using the qRT primer pairs shown in panel (a) and those for the *ACT2* mRNA as the internal standard. All values were normalized to that obtained with Col‐0; bars represent the mean (±*SD*) from three technical replicates. Letters above the bars cluster significant differences by one‐way ANOVA (*p value* <.05). (d) Growth on soil under a LD photoperiod of representative wild‐type (Col‐0), and transgenic lines expressing wild‐type SUMO1 (WT) or the SUMO1(K0) and K23,42‐R variants in the *sumo1–1 sumo2–1* background. (top) plants at flowering 40 days after sowing. (bottom) Rosettes at 20 days after sowing. Growth of the *amiS2 sumo1–1* line that suppressed *SUMO2* expression by RNAi and eliminated *SUMO1* expression by the *sumo1–1* mutation (van den Burg et al., 2010) was included for comparison. Scale bars = 2 cm.

The canonical SUMO1/2 isoforms in plants harbor seven strictly conserved lysines (K9, K10, K21, K23, K35, K41, and K42; Figure S1B) that are likely targets of secondary SUMOylation or ubiquitylation, with prior MS/MS analyses of the *Arabidopsis* SUMOylome confirming that K23 and K42 can be modified *in planta* (Miller et al., 2010, 2013). Based on our prior studies showing that these lysines are not essential for reversible conjugation at least in vitro (Augustine et al., 2016; Zhang et al., 2021), we sought to understand their impact on secondary SUMO modifications by generating *Arabidopsis* SUMO1 variants either wild type for the sequence or harboring lysine → arginine substitutions at K23 and K42 (K23,42‐R) or at all seven lysines (K0) (Figure 1a). These transgenes, expressed under the control of the native *SUMO1* promoter, were then introduced into a previously described null *sumo1–1* and *sumo2–1* background generated by T‐DNA insertional mutagenesis (Miller et al., 2010; Saracco et al., 2007).

As homozygous *sumo1–1 sumo2–1* plants are inviable by arresting growth early in embryogenesis, these transgenes were introduced by *Agrobacterium*‐mediated gene transfer into plants heterozygous for the *sumo1–1* allele and homozygous for the *sumo2–1* allele, and then screened for plants homozygous for all three loci by genomic polymerase chain reaction (PCR) of progeny from a self‐cross (Miller et al., 2010). As shown in Figure 1b,c, the wild‐type (WT) *SUMO1* and the *K23,42‐R* and *K0* mutant transgenes, and the *sumo1–1* and *sumo2–1* mutations were readily detected by genomic PCR in T2 plants generated from such self‐crosses. We subsequently confirmed presence of the transgenes by sequencing fragments amplified from genomic DNA using *SUMO1*‐specific primers (Figure 1a,b); this sequencing detected either the WT sequence or the respective lysine → arginine codon replacements. Quantitative reverse‐transcribed (q ‐sRT) PCR then identified a collection of *sumo1–1 sumo2–1* lines that expressed the WT *SUMO1* transgene or those encoding the K23,42‐R or K0 variants at levels near equal to or exceeding those from the endogenous *SUMO1/2* loci found in the Col‐0 WT background (Figure 1c).

Rescues of the lethal *sumo1–1 sumo2–1* combination by the K23,42‐R and K0 variants were immediately obvious as triple homozygous plants could be readily identified as viable and fecund as opposed to homozygous *sumo1–1 sumo2–1* embryos that abort soon after fertilization (Saracco et al., 2007). When grown under a long‐day (LD)‐photoperiod, 20‐day‐old rosettes expressing the mutant SUMOs were nearly indistinguishable from Col‐0 rosettes and *sumo1–1 sumo2–1* rosettes rescued with the WT *SUMO1* transgene (Figure 1d). Even for 40‐day‐old flowering plants, the K23,42‐R‐ and K0‐rescued lines were strikingly similar to Col‐0 and those expressing WT SUMO1, except for a slight but reproducible reduction in inflorescence height without compromising the viability of the resulting seeds. This relatively normal growth was in contrast to an *ami‐S2 sumo1–1* line first described by van den Burg et al. (2010) that is null for *SUMO1* but downregulated for *SUMO2* expression by antisense RNAi co‐suppression; it displayed stunted rosettes and dwarfed inflorescences when grown alongside the rescued mutants (Figure 1d).

### 2.2 Lysine‐null SUMO1 generates SUMO conjugates in planta

**FIGURE 2:**
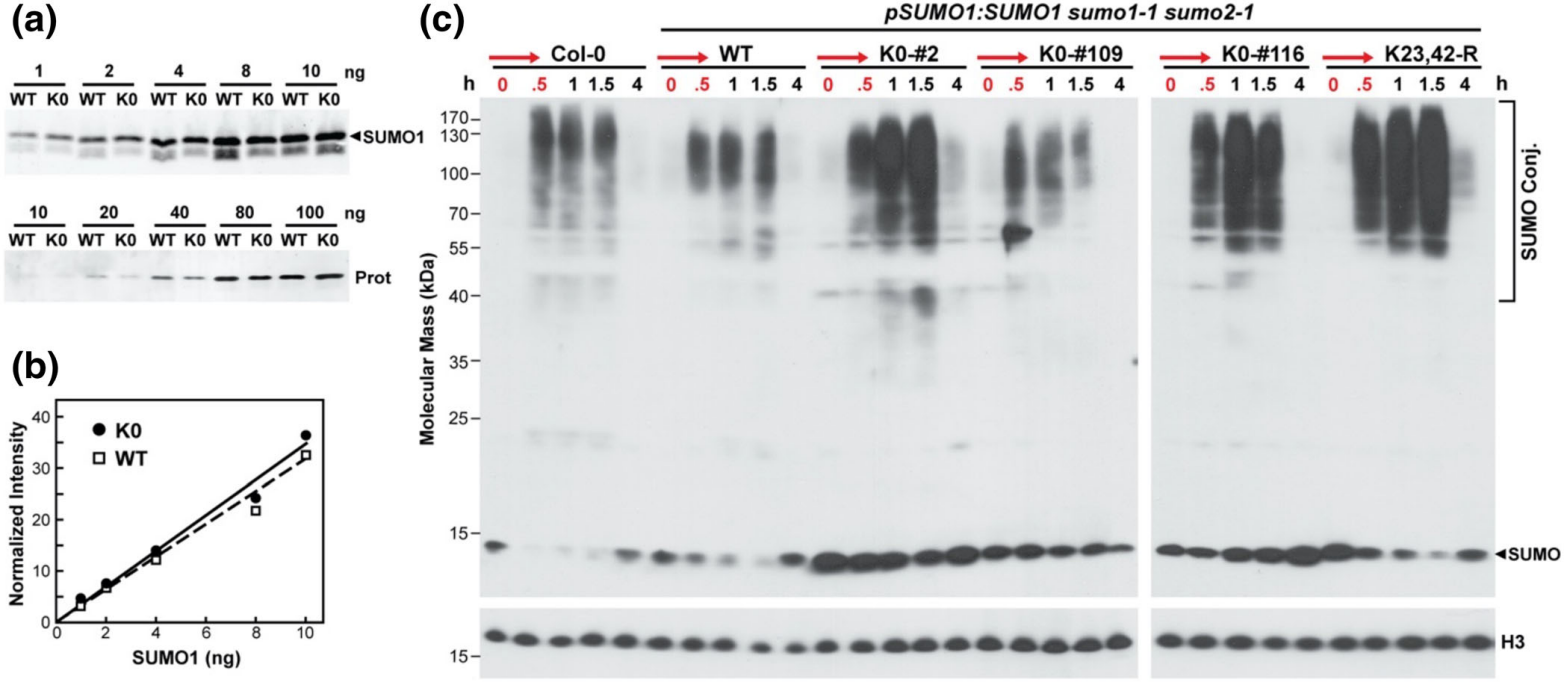
SUMO1(K0) is faithfully conjugated to *Arabidopsis* proteins during heat stress. (a) The SUMO1(K0) protein is well recognized by antibodies prepared against wild‐type (WT) *Arabidopsis* SUMO1. Increasing amounts of recombinant WT SUMO1 and SUMO1(K0) were subjected to SDS‐PAGE and immunoblot analysis with anti‐SUMO1 antibodies. (top) Immunoblot analysis with anti‐SUMO1 antibodies. (bottom) SDS‐PAGE gel stained for protein with silver. The amount (ng) applied to each lane is indicated. (b) Chemiluminescent quantification of the immunoblot signals shown in panel (a). Each point represents the mean immunoblot signal derived from three independent gel lanes as compared with those derived by protein staining. The dashed and solid lines reflect the best fit for the WT and SUMO1(K0) signal intensities, respectively. (c) Effect of heat shock on the SUMO conjugate profile in wild‐type Col‐0 seedlings, and independent transgenic *sumo1–1 sumo2–1* lines expressing either wild‐type SUMO1 (WT), or the SUMO1(K0) and SUMO1 K23,42‐R variants. Seven‐day‐old seedlings grown under continuous light in liquid medium at 24°C, were subjected at *t* = 0 to a 30‐min heat shock at 37°C (red arrows), followed by incubation at 24°C for additional times. Free SUMO and SUMO conjugates, as detected in total seedling lysates by SDS‐PAGE and immunoblotting with anti‐SUMO1 antibodies, are located by the arrowhead and bracket, respectively. Immunodetection of histone H3 was used to verify near equal protein loading.

One remarkable feature of SUMO1/2 is their robust conjugation to other proteins upon exposure of plants to acute heat stress (Augustine et al., 2016; Kurepa et al., 2003; Saracco et al., 2007). Using this response as an assay for protein functionality, we compared by immunoblot assays the SUMOylation profiles of the plants expressing the K23,42‐R and K0 variants to those expressing WT SUMO1 before and after a 37°C heat shock. As both free SUMO1/2 and their conjugates are typically detected with anti‐SUMO1 antibodies after sodium dodecylsulphate–polyacrylamide‐gel electrophoresis (SDS‐PAGE) (Kurepa et al., 2003; Saracco et al., 2007), we considered it possible that the lysine → arginine substitutions would substantially attenuate the antigenicity of the mutant polypeptides and thus artifactually depress detection of their free and conjugated forms. Fortunately, direct comparisons of antibody sensitivities against recombinant proteins revealed that the K0 variant could be detected equally well as WT SUMO1 (Figure 2a,b).

Subsequent immunoblot analyses of total extracts prepared from seedlings grown at 24°C, and then exposed to a 30‐min heat shock at 37°C followed by a return to 24°C, revealed only modest differences in the heat‐shock‐induced SUMOylome profile in *sumo1–1 sumo2–1* seedlings expressing the K23,42‐R and K0 variants as compared with Col‐0 seedlings or those expressing WT SUMO1 (Figure 2c). Even the rise and fall of conjugates with molecular mass above 50 kDa appeared mostly coincident, with low levels of conjugates evident before the heat stress (obvious in more exposed immunoblots), high levels seen after the 30 min heat shock and immediately after an ensuing re‐acclimation back to 24°C, and then a return back to low levels of conjugates after continued incubation at 24°C. In fact, all of the increases in conjugate levels were lost within 3.5 h at the cooler temperature.

An interesting feature of heat‐induced SUMOylation is that the levels of conjugates are directly proportional to the amount of free SUMO, indicating that the response is SUMO1/2 limiting (Kurepa et al., 2003; Saracco et al., 2007). This effect was also seen here for the WT and K0 transgenic lines; plants expressing high levels of SUMO1, as judged by the levels of free SUMO1/2 before the heat stress, showed strong accumulation of SUMO conjugates, while those expressing less, showed reduced conjugate accumulation (e.g., compare the WT and K0‐#116 vs. K0‐#2 and K0‐#109 lines). Taken together, we concluded that the K0 and K23,42‐R variants retained activity at least with respect to conjugation/deconjugation.

We anticipated that a block in secondary SUMO1 modifications via the lack of lysines would reduce the size distribution of SUMO conjugates in the K0 or K23,42‐R variants relative to WT SUMO1 as seen by SDS‐PAGE if such modifications are pervasive *in planta*. For example, the absence of additional SUMOs or Ubs would theoretically reduce the conjugate mass per added moiety by at least 11 and 8 kDa, respectively, which might be evident in side‐by‐side comparisons of SUMOylation profiles. As shown in Figure S2, the molecular mass distributions of both the smear of conjugates and individual species accumulating both before and after a 30‐min heat shock at 37°C were indistinguishable between Col‐0 seedlings and the *sumo1–1 sumo2–1* seedlings rescued with either WT SUMO1 or K0 transgenes. Taken together, we suggest that such secondary modifications are not prevalent in *Arabidopsis* even though they can be detected by MS/MS (Miller et al., 2010, 2013).

### 2.3 *Arabidopsis* expressing lysine‐null SUMO1 display subtle phenotypes

**FIGURE 3:**
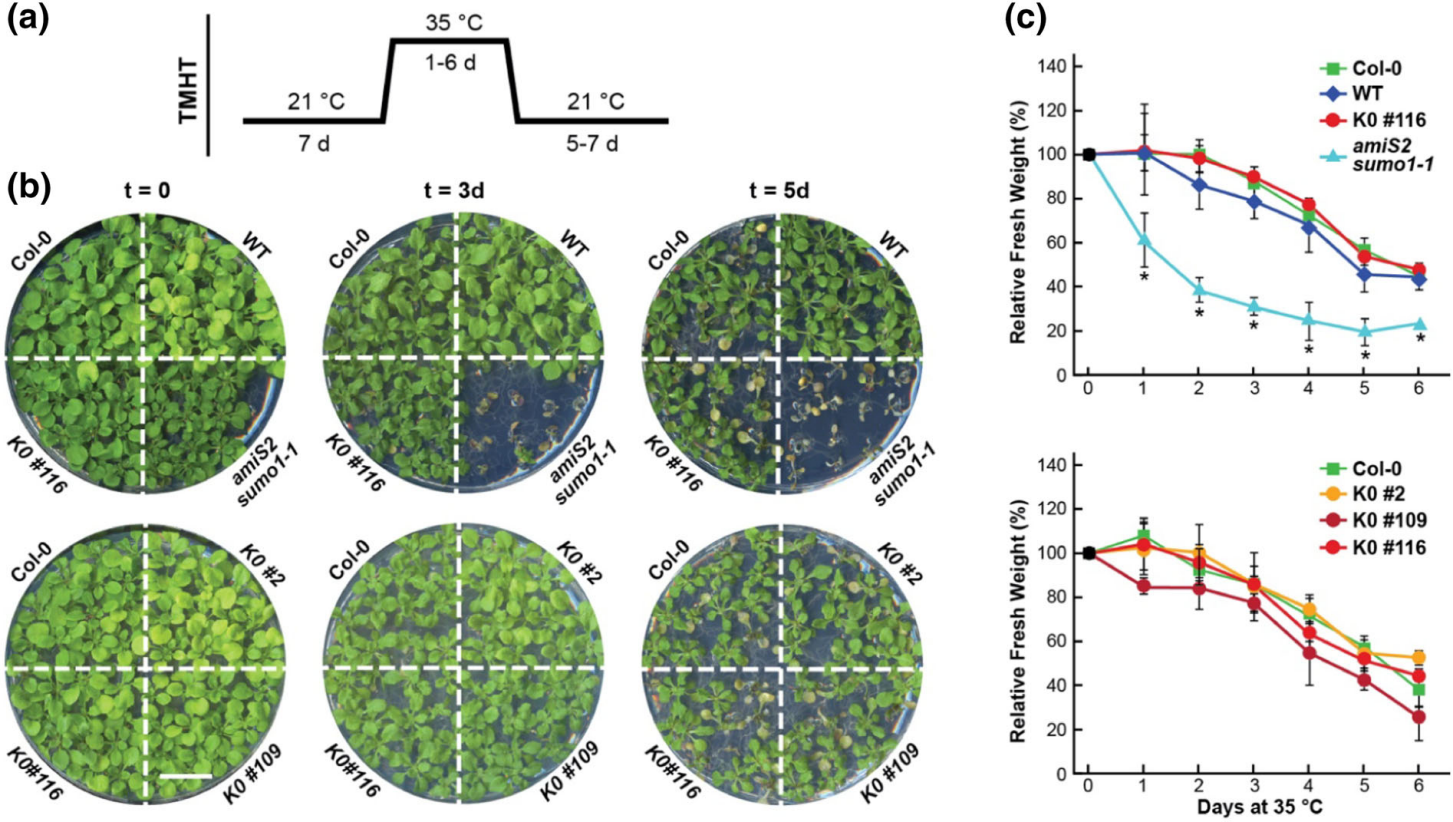
*Arabidopsis* expressing SUMO1(K0) have normal thermotolerance. (a) Treatment regimen testing the thermotolerance to moderately high temperature. Seedlings were grown under a LD photoperiod for 7 days at 21°C on solid GM medium containing 2% sucrose, incubated for 1–6 days at 35°C, and then returned to 21°C for 20 days of total growth before assay of tolerance by continued growth. (b) Representative plates of seedlings either not exposed to the heat stress (*t* = 0) or exposed to 35°C for 3 or 5 days before assay. Plates were photographed after 20 days of total growth. Shown are wild‐type Col‐0 seedlings, and homozygous *sumo1–1 sumo2–1* lines expressing under the *SUMO1* promoter either wild‐type SUMO1 (WT #101), the K23,42‐R variant, or three independent lines expressing SUMO1(K0) (K0 #2, K0 #109, and K0 #116). Survival of the *amiS2 sumo1–1* line was included for comparison. Scale bar = 1 cm. (c) Quantification of the thermotolerance assay as shown in panel (b). Plotted are the relative growth of seedlings exposed to increasing numbers of days at 35°C before return to 21°C. Each point represents the normalized mean fresh weight from three biological replicates (±*SD*). Asterisks identify significant differences as compared with Col‐0 by two‐way ANOVA (*p* value <.05).

Given the plethora of functions ascribed to plant SUMOs (Augustine & Vierstra, 2018; Benlloch & Lois, 2018; Castro et al., 2012; Morrell & Sadanandom, 2019; Yates et al., 2016), we then tested whether the K0 and K23,42‐R rescued *sumo1–1 sumo2–1* lines might impact SUMO activity more subtly, using for comparisons Col‐0 seedlings, those expressing WT SUMO1, and the *ami‐S2 sumo1–1* line as a representative of a strong but still viable mutant compromising SUMO1/2 function (van den Burg et al., 2010). Prior studies revealed that mutants in the SUMO system are hypersensitive to long‐term heat stress, which can be easily assayed by seedling growth upon prolonged exposure to moderately high temperatures (Rytz et al., 2018; Yoo et al., 2006). As shown in Figure 3, 7‐day‐old seedlings of both the K0 and the K23,42‐R lines had normal tolerances to such stress. Although the growth of *ami‐S2 sumo1–1* seedlings was substantially arrested when acclimated for 7 days at 21°C and then exposed to 35°C for as little as 1 day before returning back to 21°C, all the rescued *K0* and *K23,42‐R* seedlings showed strong heat resistance like Col‐0 seedlings or *sumo1–1 sumo2–1* seedlings rescued with WT *SUMO1*. In fact, they remained moderately viable even after a 5‐day exposure to 35°C (Figure 3).

**FIGURE 4:**
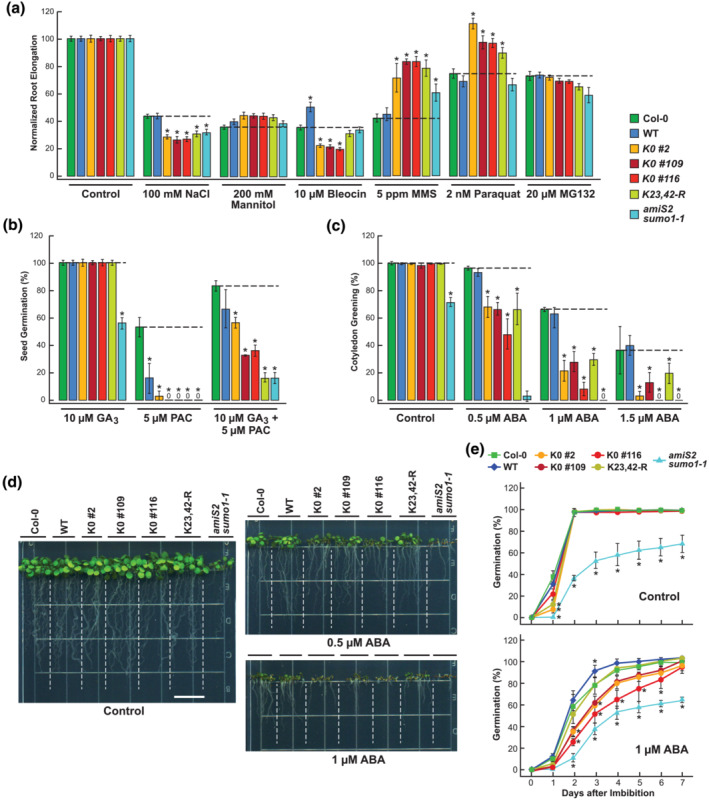
*Arabidopsis* expressing SUMO1(K0) is differentially sensitive to various stress conditions, abscisic acid (ABA), and the gibberellic acid (GA) biosynthetic inhibitor paclobutrazol (PAC). Tested were wild‐type Col‐0 and homozygous *sumo1–1 sumo2–1* seeds/seedlings expressing under the SUMO1 promoter either wild‐type SUMO1 (WT #101), the K23,42‐R variant, or three independent transgenic lines expressing SUMO1(K0) (K0 #2, K0 #109, and K0 #116). Survival of the *amiS2 sumo1–1* line was included for comparison. The dashed lines highlight the mean response of Col‐0 to each treatment condition. All percentage values were normalized to those of control seedlings without the treatment. 0 indicates treatments where no seeds germinated (panel b) or where no seedlings greened (panel c). Asterisks in panels (a, b, c, and e) identify significant differences as compared with Col‐0 by two‐way ANOVA (*p* value <.05). (a) Sensitivity of seedling growth to either 100 mM NaCl, 200 mM mannitol, 10 nM bleocin, 5 ppm methyl methanesulfonate (mMS), 2 nM paraquat, or 20 μM MG132. Seedlings were grown at 21°C on solid 1/2 MS medium for 3.5 days without the chemicals, transferred to solid medium with the indicated concentrations, and then measured for root growth after 7 additional days. Each bar represents normalized mean response (±SE). *n* = 6 for MG132 and *n* = 10 to 16 for the other treatments. (b) Sensitivity of seed germination to GA3, and the GA synthesis inhibitor PAC. Seeds were sown on solid 1/2 MS medium containing the indicated concentrations of GA and/or PAC, stratified at 4°C in darkness for 3 days, and then assayed for germination by radical protrusion following a 7‐day incubation at 21°C in white light. Each bar represents the normalized mean percentage germination of two biological replicate plates, each containing 15 seeds (±SE). (c) Sensitivity of cotyledon greening to ABA after germination. Seeds were sown on solid 1/2 MS medium containing the indicated concentrations of ABA, stratified at 4°C in darkness for 3 days, and then visually assayed for green cotyledons after 7 days of incubation under a long‐day photoperiod at 21°C. Each bar represents the normalized mean percentage of either four biological replicate plates (control, .5 μM, and 1 μM) or two biological replicate plates (1.5 μM), each containing 15 seedlings (±SE). (d) Germination and growth for 7 days of seedlings treated without or with .5‐ or 1 μM ABA. Fifteen seeds were plated for each genotype. The dashed lines roughly demarcate lanes where the roots of each line grew. Scale bar = 1 cm. (e) Percentage of seed germination for various days treated without or with 1 μM ABA. Each bar represents the normalized mean percentage of four biological replicate plates, each containing 15 seeds (±SE).

We next tested the sensitivity of the mutants to an assortment of stress‐inducing chemicals, some of which had been previously connected to the SUMO system (Benlloch & Lois, 2018; Castro et al., 2012; Morrell & Sadanandom, 2019; Xu et al., 2013; Zhang et al., 2021). The list, used at concentrations known to compromise *Arabidopsis* growth when added to solid medium, included NaCl (100 mM) for salt stress, mannitol (200 mM) for osmotic stress, the DNA‐damaging agents bleocin (10 nM) and methyl methanesulfonate (MMS; 5 ppm) that induce genotoxic stress, paraquat (2 nM) that generates cytotoxic reactive oxygen species by blocking photosynthetic electron transport, and the proteasome inhibitor MG132 (20 μM). Here, subtle but reproducible effects on seedling growth were evident for some effectors after a 7‐day treatment when comparing the K0 and K23,42‐R lines to Col‐0 and seedlings expressing WT SUMO1 (Figure 4a). Although the lysine‐less mutants grew more poorly on NaCl, they consistently grew slightly better on mannitol. Similarly, the mutants were hypersensitive to bleocin, which induces DNA strain scission, but hyposensitive to MMS, which induces DNA damage via deoxyguanosine/deoxyadenosine methylation and stalling at DNA replication forks. The DNA‐damaging agents hydroxyurea and mitomycin C were also tested but showed no differential impact on seedling growth when comparing the K0 and K23,24‐R mutants to Col‐0 even at concentrations (1 to 5 mM and 10 μg/ml, respectively) that substantially impacted seedling growth for Col‐0 (Figure S3A). The lysine‐less mutants were also less sensitive to paraquat but surprisingly only marginally impacted by MG132 despite connections of the Ub‐proteasome system to SUMO through StUbLs and the assembly of SUMO‐Ub polymers (Benlloch & Lois, 2018; Elrouby, 2015; Miller et al., 2010).

As the SUMO system has been intimately linked to plant hormone signaling (Augustine & Vierstra, 2018; Benlloch & Lois, 2018; Castro et al., 2012), we also tested the response of the K0 and K23,42‐R lines to auxin, salicylic acid (SA), abscisic acid (ABA), and gibberellic acid (GA) and its biosynthesis inhibitor paclobutrazol (PAC), all of which are influenced by SUMOylation (Conti et al., 2014; Ishida et al., 2009; Lois et al., 2003; Miura et al., 2009, 2010; Orosa‐Puente et al., 2018). Auxin and SA treatments failed to show observable differences between Col‐0 and the K0 and K23,42‐R lines using primary root elongation as the assay (Figure S3B).

Strikingly, the K0 variants were strongly hypersensitive to ABA while the K23,K42‐R lines were modestly hypersensitive, using seed germination, cotyledon greening, and seedling root growth as well‐described ABA responses, which was consistent with the known connections between ABA signaling and the SIZ1 SUMO E3 (Lois et al., 2003; Miura et al., 2009). While the K0‐lines germinated similarly to WT SUMO1 on control medium, they were substantially delayed in radical emergence and subsequent primary root elongation and cotyledon greening in the presence of ABA (Figure 4c–e). For the K0 lines, the degree of suppression roughly correlated with expression levels of the SUMO1‐K0 protein (i.e., lower levels in the K0 #109 line and higher levels in the K0 #2 and K0 #116 lines based on both qRT PCR and immunoblot assays; Figures 1c and 2c). Interestingly, the *amiS2 sumo1–1* line was even more sensitive to ABA, which nearly abolished cotyledon greening at .5 μM, and strongly delayed seed germination and root elongation at 1 μM (Figure 4c–e).

GA signaling has been linked to the SUMO system through modification of the growth repressing DELLA proteins, which are key hubs within the GA response pathway (Conti et al., 2014; Kim et al., 2015). Whereas GA sensitivity was unaltered in the K0 and K23,42‐R lines as compared with Col‐0 and the WT SUMO1 lines based on the percentage of seed germination after 7 days, it was effectively suppressed by PAC (Figure 4b). At 5 μM PAC, germination was strongly or completely suppressed in the K23,42‐R line and most of the K0 lines, while 58% and 17% of the Col‐0 and WT seeds still germinated, respectively. This suppression could be partially restored by treating the seeds with 10 μM of the bioactive GA isoform GA_3_ simultaneous with 5 μM PAC, thus confirming that the dampened seed germination was mediated by inhibited GA synthesis (Figure 4b). Taken together, it is clear that *Arabidopsis* can survive and reproduce despite a block in secondary SUMO1/2 modifications. However, we found that the lysine → arginine replacements did induce more subtle effects on plant growth and development when treated with various agents known to be influenced by SUMOylation.

### 2.4 Possible use of lysine‐null SUMOs to better describe SUMOylation

One complication in attempts to identify SUMOylated proteins and map individual SUMOylation sites by MS/MS‐based approaches is the potential complexity of the modified proteins that can bear multiple SUMOs individually attached to varying numbers of lysines within each target, each of which can be modified further with additional SUMOs and/or Ubs (Hendriks et al., 2015; Miller et al., 2010; Vertegaal et al., 2006; Wohlschlegel et al., 2006). Clearly, one way to simplify mapping of SUMOylation sites would be to employ the aforementioned SUMO1 variants bearing lysine → arginine substitution that can block such secondary modifications while also being amenable to affinity purification and MS/MS detection of SUMO binding sites (Miller et al., 2010; Vertegaal et al., 2006; Wohlschlegel et al., 2006). As shown by Miller et al. (2010, 2013), the latter is possible by replacing His‐89 in the mature SUMO1 sequence with an arginine, which then introduces a trypsin cleavage site just proximal the C‐terminal glycine. After trypsinization, SUMO binding sites can then be identified by a signature QTGG sequence isopeptide linked to the substrate lysine modified by SUMOylation. Further, by comparing MS/MS data obtained with WT SUMO and the K0 variant, mapping possible SUMO–SUMO and SUMO‐Ub linkages might be possible.

Here, we tested the feasibility of this approach by generating novel SUMO1 variants that included: (i) a N‐terminal 6His tag for affinity purification, (ii) replacement of the initiator methionine in SUMO1 with an arginine (M1R) to allow the release of the 6His sequence by trypsin and thus simplifying MS/MS mapping of potential secondary modifications sites within the flexible N‐terminal domain at Lys‐9 and Lys10 (Figure S1), (iii) replacement of all lysines with arginines (i.e., K0) to avoid secondary modifications, and (iv) termination in a H89R substitution that would introduce a C‐terminal trypsin cleavage site. The final protein sequences would enable enrichment by nickel‐nitrilotriacetic acid agarose (Ni‐NTA) resins, would effectively release upon trypsin digestion footprint fragments identifiable by MS that would bear only a signature QTGG SUMO fragment (326 m/z) isopeptide linked to a lysine remnant of the target protein, facilitate identification of these sites by reducing footprint complexity, and allow the use of LysC‐mapping PRISM strategies if needed (Hendriks et al., 2015). The latter two‐step strategy involves sequential Lys‐C and trypsin digestions of SUMOylated targets, which help focus footprint mapping to only those peptides bearing the modified lysine.

**FIGURE 5:**
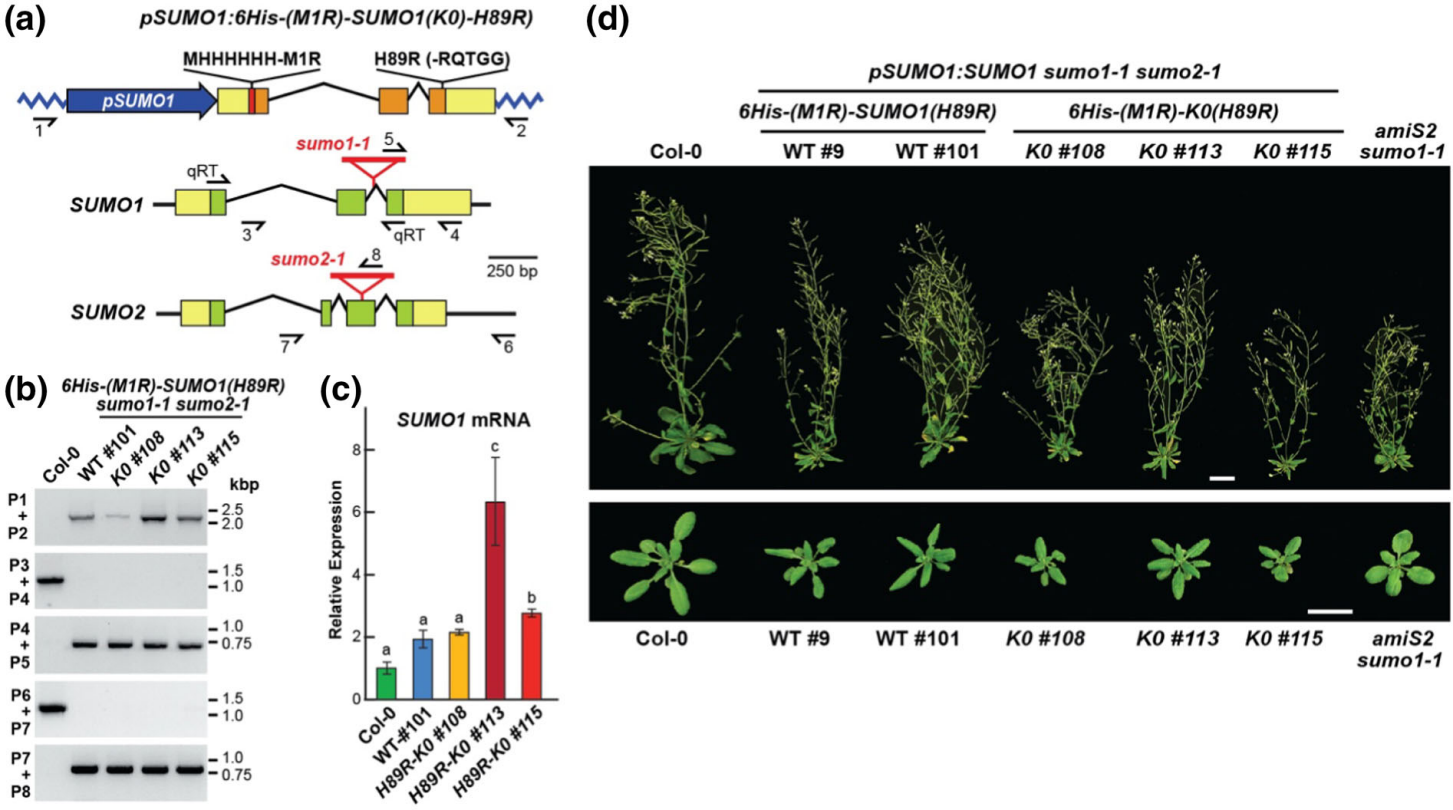
The SUMO1(K0) variant designed for mass spectrometry (MS) analysis of SUMO linkages rescues the embryo‐lethal *sumo1 sumo2* mutant. (a) Diagram of the *pSUMO1:6His‐SUMO1‐K0(H89R)* transgene expressing the *Arabidopsis* SUMO1(K0) variant designed for improved MS/MS analysis. (top) Organization of the *6His‐(M1R)‐SUMO1‐K0(H89R)* transgene. The amino acid sequences of the 6His‐(M1R) tag (MHHHHHH‐R) and the region surrounding the H89R mutation (RQTTG) are shown. (bottom) Organization of the endogenous *SUMO1* and *SUMO2* loci showing the positions of the T‐DNA insertions (red triangles) that disrupt expression. The *SUMO1* promoter is shown in blue. Untranslated and coding regions are in yellow and orange boxes, respectively. Introns are shown by broken lines. Half arrows locate the positions of the primers used for genotyping and qRT‐PCR in panels (b) and (c), respectively. (b) Genotyping of Col‐0 and the transgenic lines by PCR. Genomic DNA was isolated from 27‐day‐old wild‐type *Arabidopsis* Col‐0, and homozygous *sumo1–1 sumo1–2* seedlings expressing 6His‐(M1R)‐SUMO1(H89R) either with all seven lysines (*WT #9* and *WT #101*), or with the K0 sequence that replaced all lysines with arginines (*K0 #108*, *K0 #113*, and *K0 #115*). (c) *SUMO1* transcript levels were quantified by qRT‐PCR of total RNA extracted from 7‐day‐old seedlings, using the qRT primer pairs shown in panel (a) and those for the *ACT2* mRNA as the internal standard. All values were normalized to that of Col‐0; bars represent the mean (±*SD*) of three technical replicates. Letters above the bars cluster significant differences by one‐way ANOVA (*p value* <.05). (d) Growth on soil under a LD photoperiod of representative wild‐type Col‐0, and *sumo1–1 sumo2–1* lines expressing wild‐type 6His‐(M1R)‐SUMO1(H89R) (WT) or the 6His‐(M1R)‐K0(H89R) variant. (top) Plants at flowering (45 days after sowing). (bottom) Rosettes at 27 days after sowing. Growth of the *amiS2 sumo1–1* line was included for comparison. Scale bars = 2 cm.

Clearly, the use of such a highly engineering SUMO variant would require demonstration that it could be expressed well in plants without adverse phenotypes and be faithfully conjugated to other proteins in vivo. Toward this goal, we generated *6His‐(M1R)‐SUMO1(H89R)* transgenes harboring either WT *SUMO1* or the *K0* variant expressed under the control of the native *SUMO1* promoter (Figure 5a), which were then transformed into *Arabidopsis* plants heterozygous for the *sumo1–1* allele and homozygous for the *sumo2–1* allele. As shown in Figure 5b,c, genomic PCR and qRT‐PCR screens of T2 progeny from a self‐cross succeeded in identifying triple homozygous plants expressing either transgene. Remarkably, despite all the modifications to the SUMO1 template, the rescued plants developed reasonably normal rosettes after 27 days growth under a LD photoperiod, and fertile inflorescences after 47 days (Figure 5d). The main developmental differences as compared with Col‐0 were a modest reduction in rosette size and shorter inflorescences, which resembled somewhat those of the *ami‐S2 sumo1–1* mutant (Figure 5d). Interestingly, the slightly aberrant phenotypes seen for the *6His‐(M1R)‐K0(H89R)* lines were indistinguishable from those for the *6His‐(M1R)‐SUMO1(H89R)* lines, suggesting that the observed defects were primarily caused by the 6His(M1‐R) and H89R additions and not by the K0 lysine → arginine substitutions (Figure 5d).

**FIGURE 6:**
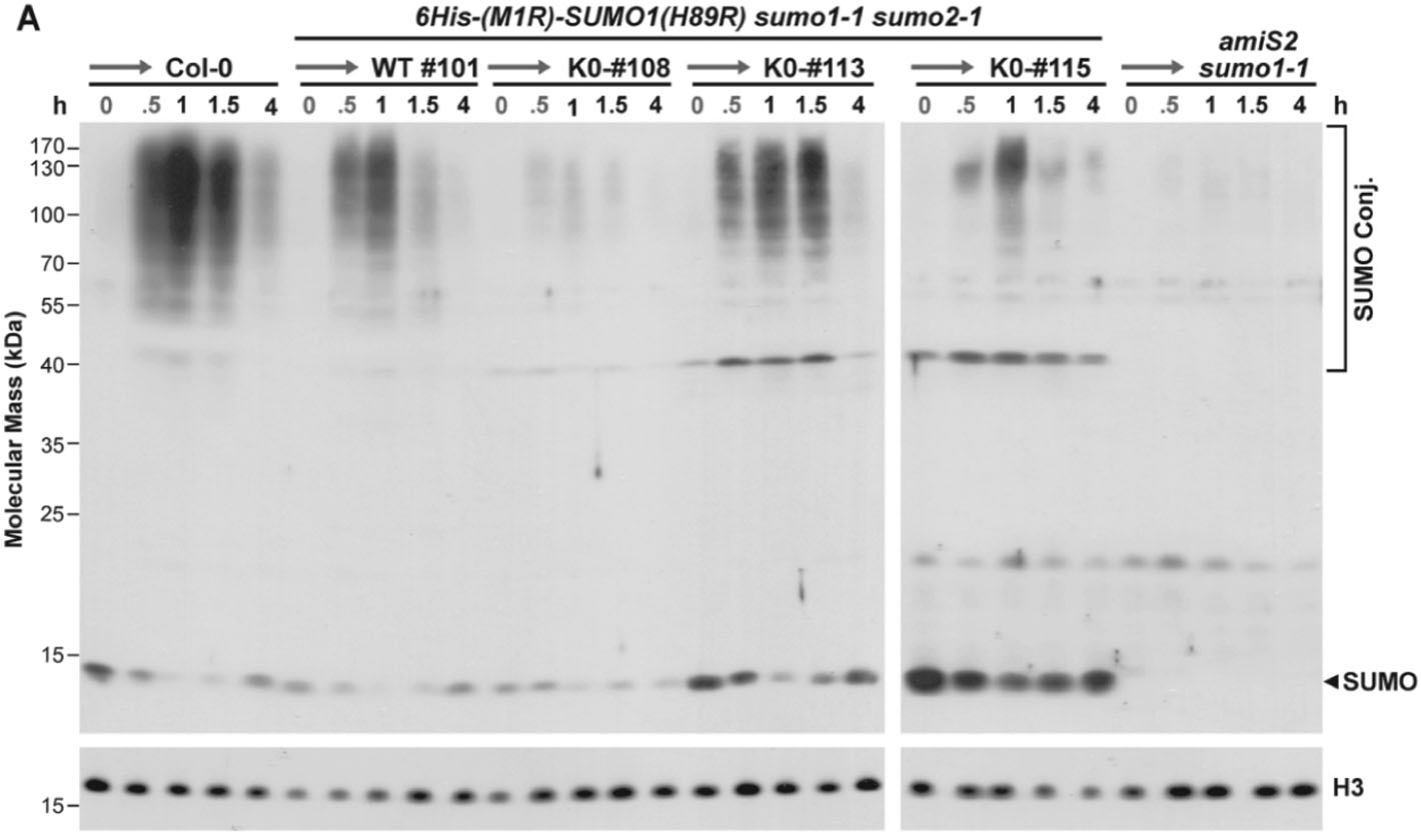
6His‐(M1R)‐SUMO1‐K0(H89R) faithfully conjugates to *Arabidopsis* proteins during heat stress. Seven‐day‐old seedlings, either wild type (Col‐0) or harboring the *sumo1–1 sumo2–1* mutations together with the *6His‐(M1R)‐SUMO1(H89R)* and *6His‐(M1R)‐K0(H89R)* transgenes were grown under continuous light in liquid MS medium at 24°C, subjected to a 30 min heat shock at 37°C (red arrows), and then returned back to 24°C for additional times. Total protein extracts were subjected to SDS‐PAGE and immunoblot analysis with anti‐SUMO1 antibodies. Free SUMO1 and SUMO conjugates are located by the arrowhead and brackets, respectively. Immunoblot analysis with anti‐histone H3 antibodies was used to verify near equal protein loading.

We next tested whether the 6His‐(M1R)‐SUMO1(H89R) and 6His‐(M1R)‐K0(H89R) proteins retained functionality as judged by their reversible conjugation to other proteins *in planta*, using a 37°C heat shock to stimulated SUMOylation. As compared with Col‐0, *sumo1–1 sumo2–1* lines expressing either SUMO1 variant readily generated conjugates with other proteins soon after the 30‐min heat stress, with the conjugates then dissembled concomitant with the reappearance of free SUMO1 after the seedlings were returned back to 24°C (Figure 6). By in large, the size distribution and kinetics of conjugate assembly/disassembly for the SUMO1 and K0 variants were also indistinguishable when comparing the WT #101 and K0 #113 lines (Figure 6). However, we presume that the SUMO1 variant proteins are probably not completely WT as judged by their dampened assembly into conjugates based on their relative levels versus free SUMO1 seen during the heat shock (Figure 6).

**FIGURE 7:**
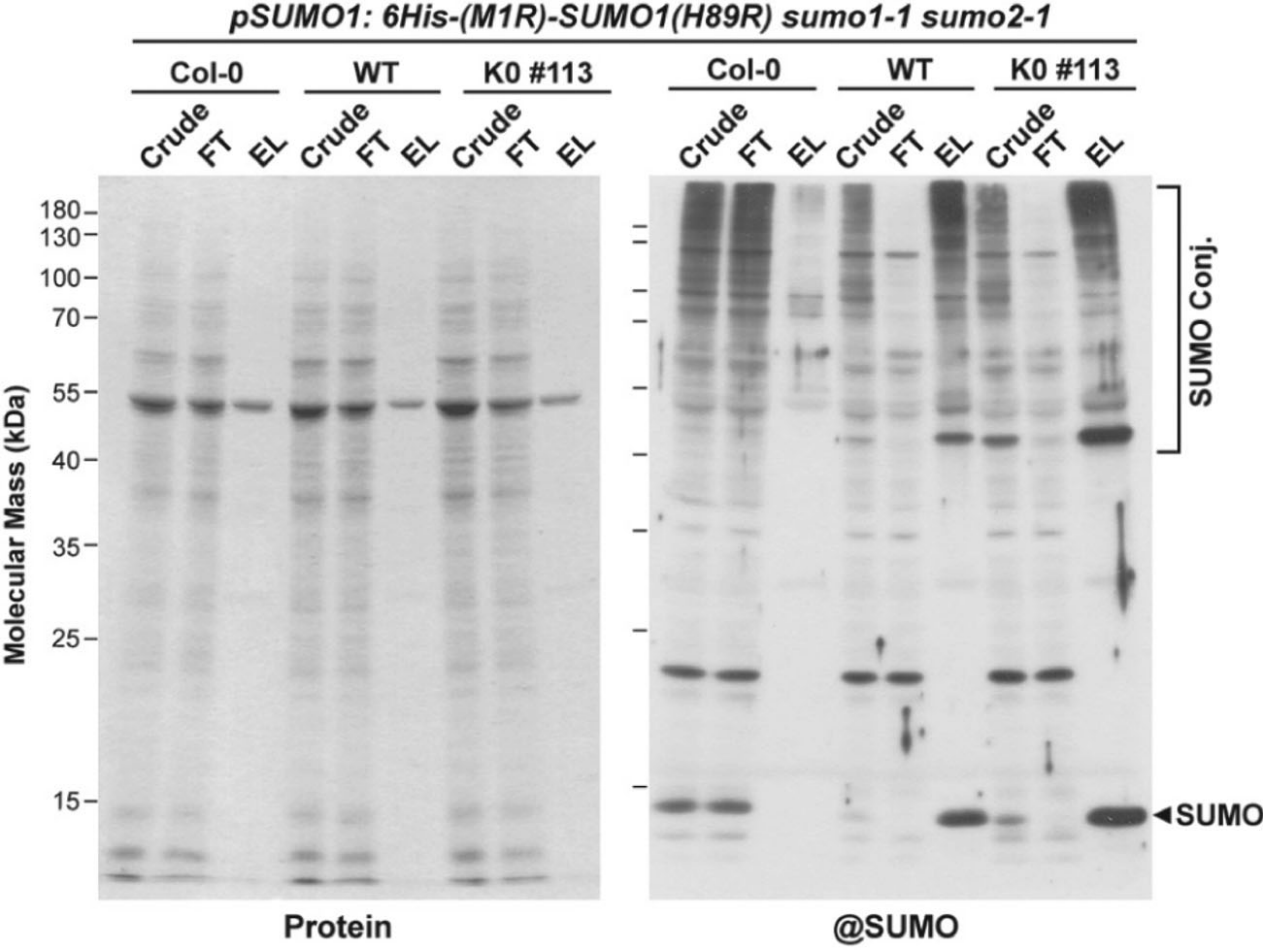
Use of an engineering variant of SUMO1 to enrich for SUMOylated proteins from *Arabidopsis*. Col‐0 wild‐type seedling or *sumo1–1 sumo2–1* seedlings expressing the *6His‐(M1R)‐SUMO1(H89R)* variant encoding either wild‐type SUMO1 (WT) or the K0 variant were grown for 7 days in liquid culture followed by a 30‐min heat shock at 37°C. The seedlings were then homogenized and subjected to Ni‐NTA affinity chromatography. Equivalent protein amounts from the crude extract, flow‐through (FT) or eluate (EL) fractions were subjected to SDS‐PAGE and either stained for protein with Coomassie blue (left panel) or subjected to immunoblot analysis with anti‐SUMO1 antibodies (right panel). Free SUMO1 and SUMO conjugates are located by the arrowhead and brackets, respectively.

As a prelude to more in depth proteomic analysis of SUMOylated proteins, we then tested whether these engineered SUMO1 variants could be exploited to affinity purify SUMOylated proteins directly from *Arabidopsis*. As shown in Figure 7, a single Ni‐NTA chromatography step was sufficient to enrich for SUMOylated species from heat‐shocked seedlings as judged by protein staining and immunoblot analysis with anti‐SUMO1 antibodies. Although most seedling proteins in all backgrounds flowed through the Ni‐NTA column, SUMOylated proteins were selectively retained (as judged by immunoblotting) when using the *6His‐(M1R)‐SUMO1(H89R)* and *6His‐(M1R)‐K0(H89R)* lines as compared with Col‐0.

We next analyzed the eluate fractions by LC–MS/MS after trypsinization and found that the engineered lines could be used to identify SUMOylated proteins. While preliminary, we found that previously known SUMOylated proteins could be specifically detected in eluates from *6His‐(M1R)‐SUMO1(H89R)* and *6His‐(M1R)‐K0(H89R)* plants as compared with that from Col‐0 (Table S2). Notable examples from the list of 109 potential substrates, many of which were identified by us previously (Miller et al., 2010, 2013; Rytz et al., 2018), included the alteration/deficiency in activation (ADA)‐2A/ADA2B transcriptional coactivators, histone deacetylase (HDA)‐19, ethylene‐insensitive (EIN)‐3, the trihelix global transcription factor GT‐2, ribosomal small subunit protein (RPS)‐3, the histone demethylase IBM1, the PICKLE chromatin remodeler, the SEUSS transcriptional regulator, the SAE2 subunit of the SUMO E1 activating enzyme, the TOPLESS‐related‐1 co‐repressor, and the WRKY transcription factor‐3 (Table 1). Insomuch that these results demonstrated the applicability of the transgenic lines, it is clear that further enrichment of conjugates by a second step would be required for more stringent, in‐depth MS/MS studies (e.g., Hendriks et al., 2015; Miller et al., 2010, 2013; Rytz et al., 2018).

**TABLE 1:** Proteins identified by tandem mass spectrometry with 6His‐(M1R)‐SUMO(H89R) harboring WT and K0 SUMO1^a^.

Protein^b^	Accession number	Description
ACO2	At1g62380	ACC oxidase‐2
ADA2A	At3g07740	ADA2A transcriptional coregulator
ADA2B	At4g16420	ADA3B transcriptional coregulator
ANAC12	At5g63790	NAC transcription factor‐12
BRM	At2g46020	SNF2 transcriptional regulator
DRIP2	At2g39580	DREB2A‐interacting protein‐2
EIN3	At3g20770	Ethylene‐insensitive‐2
EML3	At5g13020	ENT/Tudor transcriptional regulator‐3
GT‐2	At1g76890	Global transcription factor‐2
HDA19	At4g38130	Histone deacetylase‐19
HAG1	At3g54610	GNAT histone acetyltransferase‐1
HSFA1A	At4g17750	Heat shock transcription factor‐A1A
HSP81–2	At5g56030	Heat shock chaperone‐81A
IBM1	At3g07610	Jumonji transcription factor
IDN2	At3g48670	Involved‐in de novo RNA methylation‐2
MED23	At1g23230	Mediator complex subunit‐23
MED25	At1g25540	Mediator complex subunit‐25
OBAP2A	At5g45690	Histone acetyltransferase
PKL	At2g25170	Pickle chromatin remodeler
RBR1	At3g12280	Retioblastoma‐Related‐1
RGD3	At3g54280	Ribosomal subunit‐3C
RSP3C	At5g35530	Root growth defective‐3C DNA‐binding
SAE2	At2g21470	SUMO‐activating Enzyme‐2
SDN3	At5g67240	Small RNA‐degrading nuclease‐3
SEU	At1g43850	SEUSS transcriptional regulator
SGR6	At3g48550	Shoot gravitropism‐like‐6
SLK1	At4g25520	SEUSS‐like transcriptional regulator‐1
SNL2	At5g15020	SIN3‐Like‐3 transcriptional repressor‐2
SUVR1	At1g04050	Histone N‐methyltransferase‐1
SWI3C	At1g21700	Sucrose non‐fermenting‐3C
SYN4	At5g16720	Sister chromatin cohession‐1 protein‐4
TCP9	At2g45680	TCP transcription factor‐9
TRP1	At1g80490	Topless‐related‐1
TRO	At1g51450	TRAUCO trithorax embryogenesis regulator
WRKY3	At2g03340	WRKY transcription factor‐3

^a^ List highlights 35 proteins from the full catalog of 109 proteins selectively identified by tandem mass spectrometry (MS/MS) analysis of proteins enriched from *6His‐(M1R)‐SUMO1(H89R)* and *6His‐(M1R)‐K0 (H89R)* plants compared with Col‐0 wild type.

^b^ Proteins colored in red or their paralogs were identified in previous MS/MS analyses of SUMOylated proteins from *Arabidopsis* (Miller et al., 2010, 2013; Rytz et al., 2018).

## 3 DISCUSSION

Although accumulating evidence in yeasts and mammalian cells revealed important roles for secondary modifications of SUMO either by additional SUMOylation or by ubiquitylation (Keiten‐Schmitz et al., 2019; Vertegaal, 2010), their possible functions in plants have remained obscure, except for the detection of such modifications by MS/MS (Miller et al., 2010, 2013) and by preliminary genetic studies on potential *Arabidopsis* StUbLs that were presumed to ubiquitylate SUMOs based on sequence homology (Elrouby et al., 2013; Hale et al., 2016). Here, we attempted to directly address this question through complementation studies that combined a mutant eliminating expression of the two related and essential SUMOs in *Arabidopsis* (SUMO1 and 2) with a SUMO1 variant in which all lysines were replaced with arginines. Prior studies with this SUMO1(K0) protein showed that it is immune to secondary SUMOylation at least in vitro using purified components (Augustine et al., 2016).

Surprisingly, these *SUMO1(K0) sumo1–1 sumo2–1* plants were viable and relatively normal phenotypically when grown under optimal conditions, and showed similar thermotolerance to moderately high temperatures and indistinguishable SUMOylation patterns both before and after a strong heat shock of 37°C. Both the rates of conjugate assembly and disassembly were relatively normal, indicating that the E1 → E2 → E3 conjugation cascade and the deSUMOylating enzymes in general can tolerate the lysine → arginine replacements. A similar set of normal responses were also seen for the K23,K42‐R replacement affecting the only lysine modification sites detected thus far *in planta* (Miller et al., 2010). The SDS‐PAGE profiles of K0‐ and K23,42‐R‐derived conjugates also appeared unaltered as compared with those of endogenous SUMOs or ectopically expressed WT SUMO1, suggesting either that few *Arabidopsis* proteins undergo secondary SUMO–SUMO or SUMO‐Ub modifications at these lysines or that their apparent molecular masses are insufficiently impacted by the additional SUMOs or Ubs to be seen electrophoretically. Consequently, we are left to propose that secondary modifications of SUMO are relatively minor even if they do occur *in planta*, as compared with direct modification of substrates through isopeptide addition at one or more substrate lysines. In fact, it is possible that SUMOylation of SUMO1/2 seen previously with *Arabidopsis* (Miller et al., 2010, 2013) represents an overzealous conjugation machinery analogous to that proposed for the SUMO ‘spray’ modification of proteins near the directly intended target (Psakhye & Jentsch, 2012).

The one caveat to our conclusions is the possibility that the non‐canonical SUMO isoforms (SUMO3 and 5) could replace the lysine‐less SUMO1 functionally as well as in forming SUMO–SUMO and SUMO‐Ub secondary modifications, but this seems unlikely given that neither endogenous loci can rescue *sumo1–1 sumo1–2* backgrounds (Saracco et al., 2007), while the SUMO1(K0) mutant provides strong replacement (this report). Another scenario, also unlikely, is that other residues beside lysine can provide secondary attachment sites in plants. While no data yet exist for SUMO, accumulating evidence with ubiquitylation in mammalian cells have discovered that other amino acids can be linkage sites. The best understood is the N‐terminal methionine amino group, which for Ub has been found to be a peptide‐bond acceptor site for concatenating linear poly‐Ub chains through the LUBAC E3 ligase complex involved in immune signaling, xenophagy, and protein quality control (Rodriguez Carvajal et al., 2021). Additional Ub acceptor sites include cysteine through thioester bonds and serine and threonine through oxyester bonds (McClellan et al., 2019; McDowell & Philpott, 2013). To date, none of these non‐canonical linkages for Ub, nor any alternative SUMO linkage site besides lysines, have been detected in plants. Nor have plant orthologs of LUBAC been found.

Despite having little influence on *Arabidopsis* growth and development under optimal conditions, expression of the SUMO1(K0) variant does elicit a variety of more subtle growth defects when exposed to various stress conditions and hormones, some of which have been linked to SUMOylation through prior genetic analyses of mutants within the SUMO system (Benlloch & Lois, 2018; Castro et al., 2012; Conti et al., 2014; Kim et al., 2015; Lois et al., 2003; Miura et al., 2009; Morrell & Sadanandom, 2019; Xu et al., 2013; Zhang et al., 2021). Included are a hypersensitivity to DNA damaging agents, a differential sensitivity to salt and maybe osmotic stress, and a hypersensitivity to ABA and the GA‐biosynthesis inhibitor PAC. Although it is possible that these effects were caused by a block in specific secondary SUMO modifications that have restricted phenotypic consequences, it is also possible that the arginine replacements selectively impact only a few SUMOylation/deSUMOylation events by directly altering interactions with downstream partners (e.g., proteins with SIMs) either before or after attachment. Whatever their roles, the lysine → arginine replacements appear relatively inconsequential to *Arabidopsis* embryo development and seedling growth.

Given that lysine‐null SUMOs are functional in plants, the possibility arises that such variants might be valuable in identifying SUMOylated proteins and their attachment sites by simplifying SUMOylation profiles and footprint analysis. Here, we provided preliminary evidence that such alterations could be useful using a further engineered SUMO1 variant with or without the K0 modifications. This variant harbors an N‐terminal 6His tag for purification, replacement of Met‐1 with an arginine to simplify MS analyses by enabling removal of the 6His sequence with trypsin, and introduction of an arginine at residue 89 thus introducing a SUMO footprint recognizable as a SUMO lysine isopeptide modified with QTGG sequence (326 m/z) that is distinct from ubiquitylation (116 m/z) (Miller et al., 2010, 2013). As shown here, *Arabidopsis* can also tolerant these additional changes by the ability of these transgenes to rescue inviable *sumo1–1 sumo2–1* plants, and by their relatively normal phenotype and SUMOylation patterns before and after heat stress. However, the plants displayed a subtle dwarf phenotype for their rosettes and inflorescences and less heat shock‐induced SUMOylation, indicating that the modified SUMO1 is not completely normal. Comparisons between the WT and K0 variants suggest that these effects were caused by the N‐ and C‐terminal changes (6His‐[M1R] and [H89R]) and not by the absence of lysines.

Preliminary studies showed that these 6His‐(M1R)‐SUMO1(H89R) and 6His‐(M1R)‐K0(H89R) proteins not only enabled enrichment of SUMO conjugates by Ni‐NTA chromatography but also allow their detection by MS/MS analysis of the samples. As seen previously (Augustine & Vierstra, 2018; Miller et al., 2010, 2013; Rytz et al., 2018), many in the catalog of conjugates have direct links to various nuclear functions, including transcription, epigenetic modifications, chromatin structure, and RNA metabolism. However, we acknowledge that additional enrichment steps, possibly using anti‐SUMO affinity chromatography (Miller et al., 2010) or the LysC‐based PRISM strategy (Hendriks et al., 2015; Miller et al., 2010), will likely be required to generate samples suitable for detailed proteomic analysis of SUMOylation and the mapping to SUMO1/2 attachment sites. Clearly, these engineered lines should provide valuable new tools for continued investigations of the plant SUMOylome.

## 4 EXPERIMENTAL PROCEDURES

### 4.1 Plant materials and growth conditions

The *Arabidopsis thaliana* ecotype Columbia (Col‐0) was used as the genetic background for all plant lines. The *amiS2 sumo1–1* line was as described (van den Burg et al., 2010). Prior to phenotypic studies, all the mutant and transgenic lines were grown simultaneously and the resulting seeds were allowed to after ripen for 2 months at 21°C to establish uniform seed populations with high germination rates. Unless otherwise noted in the figure legends, seeds were surface sterilized using the vapor‐phase method and stratified in water at 4°C for 2 days in the dark before germination on MS medium (1/2 MS medium supplemented with 1% sucrose and .05% MES [pH 5.7]). For samples used for immunoblot analysis and proteomics, seedlings were grown under continuous white light irradiation for 8 days at 24°C in liquid 1/2 MS medium followed by further incubation at 24°C or 37°C before harvest. For response to inhibitors, seedlings were grown on solid 1/2 MS medium containing 1% sucrose and 1% agar at 21°C under a LD photoperiod (16‐h light/8‐h dark) for 3.5 days. Seedlings of approximately the same size were transferred onto fresh 1/2 MS plates containing 100 mM NaCl, 200 mM mannitol, 10 nM bleocin (Sigma), 5 ppm methyl methanesulfonate (MMS) (Sigma), 2 nM paraquat (*N*,*N*′‐dimethyl‐4,4′‐bipyridinium dichloride, Sigma), or 20 μM MG132 *(N*‐benzyloxycarbonyl‐L‐leucyl‐L‐leucyl‐L‐leucinal; Selleckchem), and the tips of the roots were recorded. Further root growth during 7 additional days was measured from three biological replicates, each containing 15 seedlings. For MG132 treatments, an equal volume of DMSO was added to control plates. For thermotolerance assays, seedlings were grown for 7 days at 21°C under a LD photoperiod on solid GM medium containing 2% sucrose, transferred to 35°C for various days, and then were allowed to recover at 21°C (Rytz et al., 2018). Seedlings were imaged and fresh weights were measured at 20 days.

For germination assays, the seeds were sown onto solid 1/2 MS medium containing the indicated concentrations of ABA, GA3, or PAC (all from Sigma). Percentage of germination as measured by radical emergence was recorded daily for 7 days. Cotyledon greening was assessed visually by recording the percentage of seedlings with green cotyledons after 7 days. For the ABA treatments, most measurements represented the mean of four biological replicates. The GA and PAC treatments recorded two biological replicates.

### 4.2 Construction of the K0‐SUMO1 transgenic lines

Transgenic lines rescuing the homozygous *sumo1–1 sumo2–1* double mutant with WT or mutant *SUMO1* transgenes were created as described by Saracco et al. (2007). In detail, a genomic *SUMO1* fragment beginning 979‐bp upstream sequence of the start codon and ending 269‐bp downstream of the stop codon was PCR amplified from genomic DNA, using the primer pair CACCGGTTATTCTCGAGTGTATCTTCAGAAACAG and CAATAATTAATAGCTTTTTACC‐GTTACCATACCAACAAAC, and inserted into the pDNR221 gateway donor vector (Thermo Fisher Scientific). Codons for the seven lysines were sequentially converted to those for arginines by QuikChange (StrateGene) mutagenesis combined with dedicated primers. See Table S1 for the list of oligonucleotide primers. Gateway LR recombination reactions (Thermo Fisher Scientific) introduced the genomic *SUMO1* or *K0‐SUMO1* constructions into the pMDC99 plant transformation vector (Curtis & Grossniklaus, 2003), which was then inserted by the floral‐dip method into plants heterozygous for *sumo1–1* (SAIL_296_C12) and homozygous for *sumo2–1* (Salk_129775) (Saracco et al., 2007). T1 seedlings harboring the *SUMO1* and *K0* transgenes were identified by hygromycin resistance and confirmed by genomic PCR using the EconoTaq Plus Green 2X MasterMix (Lucigen). Selected transformants were allowed to self‐cross and T2 seedlings were screened for the *SUMO1* transgenes and for homozygosity of the *sumo1–1* and *sumo2–1* alleles by hygromycin resistance followed by genomic PCR using primers located in Figure 1a. Primers P1 and P2 were used to detect the 2.3‐kb transgene. Primers P3, P4 and P5 were used to detect the *sumo1–1* allele, and the P6, P7, and P8 primers were used to detect the *sumo2–1* allele.

To construct the *6His‐(M1R)‐SUMO1(H89R)* and *6His‐(M1R)‐K0(H89R)* lines, the *SUMO1* promoter and *SUMO1* coding region plus 3’‐UTR were amplified separately from the *SUMO1* genomic fragment described above with primers designed to add an *Xba*I restriction site to the 3′ end of the promoter and an *Xba*I site followed by the codons that replaced the initiator methionine codon with the MHHHHHHR protein sequence (6His‐[M1R]). The *6His‐SUMO1* construction was inserted into the *Xba*I site of the *pDNR221‐SUMO1* vector, which was then modified to harbor the H89R codon variant as described (Miller et al., 2010). Codons for the N‐terminal methionine and the six remaining internal lysines were converted to those for arginines by QuikChange as above. These constructions were introduced into the pMDC100 plant transformation vector by Gateway LR recombination reaction (Curtis & Grossniklaus, 2003) and then inserted into heterozygous *sumo1–1*, homozygous *sumo2–1* plants as described above. T1 seedlings harboring the *6His‐(M1R)‐SUMO1(H89R)* and *6His‐(M1R)‐K0(H89R)* transgenes were identified by kanamycin resistance followed by genomic PCR and allowed to self‐cross to generate T2 plants homozygous for the *sumo1–1, sumo2–1*, and transgene loci as determined by genomic PCR.

### 4.3 qRT‐PCR analyses

Total RNA was extracted from 7‐day‐old seedlings using the RNeasy Plant Mini Kit (Qiagen) followed by first strand synthesis with *oligo* (dT) primers using the SuperScript III First‐Strand Synthesis System (Invitrogen‐Thermo Fisher Scientific). qPCR was performed with a BioRad CFX Connect Real‐Time System together with the LightCycler 480 SYBR Green I Master mix (Roche). Abundance of the *SUMO1* transcript was normalized to that generated with *ACT2* based on the comparative threshold method (Pfaffl, 2001).

### 4.4 Immunoblot analyses and antigenicity test

For SUMO1/2 immunodetection, seedlings were grown in liquid 1/2 MS medium for 7 days at 24°C, heat‐shocked at 37°C for 30 min, and then returned for 24°C for various times. Tissue was blotted dry, flash frozen in liquid nitrogen, pulverized by a mortar and pestle, and mixed with two volumes (μl) per milligrams of fresh weight of twice‐strength SDS‐PAGE sample buffer (125 mM Tris–HCl, 3% SDS, 20% glycerol, and .01% bromophenol blue). After heating for 5 min to 100°C, the samples were clarified at 16,000 *g* for 10 min, subjected to SDS‐PAGE, and electrophoretically transferred onto Immobilon‐P PVDF membranes (EMD‐Millipore). The membranes were blocked for 30 min with 5% non‐fat dry milk in phosphate‐buffered saline (140 mM NaCl and 10 mM Na_2_PO_4_ [pH 7.4]) supplemented with .2% Tween‐20, and probed with rabbit anti‐*Arabidopsis* SUMO1 antibodies (Abcam, Cat. No. ab5316) as described (Kurepa et al., 2003). Rabbit antibodies against the histone‐3 (Abcam, Cat. No. ab1791) were used to confirm equal loading. The membranes were probed with horse‐radish peroxidase‐decorated goat anti‐rabbit secondary antibodies (SeraCare, Cat. No. 5220‐0341) used at a dilution of 1:10,000, followed by chemiluminescence signal detection with the SuperSignal West Pico Plus Chemiluminescent Substrate (Thermo Fisher Scientific) together with X‐ray film.

For the antigenicity tests of the anti‐SUMO1 antibodies, fragments encoding recombinant WT 6His‐SUMO1 and 6His‐SUMO1(K0) in their active forms (i.e., ending in the di‐Gly motif) were inserted into the pDEST17 plasmid (Thermo Fisher Scientific) and expressed in *Escherichia coli* Rosetta (DE3)pLysS cells (EMD Millipore) by overnight induction with isopropyl ß‐D‐1‐thiogalactopyranoside (Sigma), followed by purification using Ni‐NTA beads (QIAGEN). Antigenicity was quantified by immunoblot analysis of serial dilutions of the purified proteins with anti‐SUMO1 antibodies followed by detection with IRDye 800CW goat anti‐rabbit antibodies (LI‐COR). Signals were quantified with a LICOR Odyssey Classic FluorImager set at the 800‐nm channel, which were normalized against background by the LICOR imaging software.

### 4.5 Affinity purification of SUMO conjugates

Seven‐day‐old seedlings cultured in the above‐mentioned liquid MS medium were heat‐shocked at 37°C for 30 min, blotted dry, and immediately frozen in liquid nitrogen. Approximately 25 g of frozen tissue was pulverized and resuspended at 55°C in extraction buffer (EXB; 100 mM Na_2_HPO_4_, 10 mM Tris–HCl, 300 mM NaCl, and 10 mM iodoacetamide [IAA]) and made 7 M guanidine‐HCl, 10 mM sodium metabisulfate, and 2 mM PMSF just before use (final pH 8.0) (Rytz et al., 2016). The extract was filtered through two layers of Miracloth (EMD Millipore), clarified by centrifugation at 15,000 *g*, and incubated overnight at 4°C with Ni‐NTA resin (Qiagen) (.75‐ml resin/5 g of tissue) after addition of imidazole to 10 mM. The Ni‐NTA beads were washed sequentially with 10 column volumes of EXB containing 6 M guanidine‐HCl, .25% Triton X‐100, and 10 mM imidazole (pH 8.0); 10 column volumes of EXB containing 8M urea, .25% TritonX‐100, and 10 mM imidazole (pH 6.8); and 15 column volumes of EXB containing 8 M urea, .25% Triton X‐100, and 10 mM imidazole (pH 8.0). SUMO conjugates were released with five column volumes of elution buffer containing 350 mM imidazole, 100 mM Na_2_HPO_4_, 10 mM Tris–HCl, and 10 mM IAA (pH 8.0). The eluant was concentrated by ultrafiltration with a 10‐kDa molecular mass cutoff filter (Vivaspin; GE Healthcare Life Sciences).

### 4.6 MS/MS analysis of SUMO conjugates

The Ni‐NTA‐eluted fractions were processed for MS/MS analysis as described (Rytz et al., 2018). Samples were reduced and alkylation with 20 mM IAA with the reaction quenched with 20 mM dithiotreitol, and then diluted with 25 mM ammonium bicarbonate. The samples were digested overnight at 37°C with .5 μg of sequencing‐grade modified porcine trypsin (Promega), lyophilized to a final volume of ~250 μl, acidified with .5% (v/v) trifluoroacetic acid (pH < 3.0), and desalted and concentrated using a 100 μl Pierce C18 pipette tip (Thermo Fisher Scientific). Bound peptides were eluted in 50 μl of 75% acetonitrile and .1% acetic acid, lyophilized, and resuspended in 20 μl 5% acetonitrile and .1% formic acid.

The peptides were analyzed with a Q‐Exactive Plus mass spectrometer (Thermo Fisher Scientific) after reversed‐phase nano‐high‐performance liquid chromatography separation over 135 min with a 25 cm analytical C18 resin column (Acclaim PepMap RSLC; Thermo Fisher Scientific) and a 5%–95% acetonitrile gradient in .1% formic acid at a flow rate of 250 nl/min (Rytz et al., 2018). Data‐dependent acquisition of full MS scans (mass range of 380–1500 m/z) at a resolution of 70,000 was collected, with the automatic gain control target set to 3 × 10^6^ and the maximum fill time set to 200 ms. High energy collision‐induced dissociation fragmentation of the 15 strongest peaks was performed with an intensity threshold of 4 × 10^4^ counts and an isolation window of 3.0 m/z and excluded precursors that had unassigned, +1, +7, +8 or > +8 charge states. MS/MS scans were conducted at a resolution of 17,500, with an automatic gain control target of 2 × 10^5^ and a maximum fill time of 100 ms.

The resulting datasets were queried by Proteome Discoverer (version 2.0.0.802; Thermo Fisher Scientific) against the *A. thaliana* proteome database Araport11 (http://www.arabidopsis.org/download/) and a list of common protein contaminants (McLoughlin et al., 2018). Peptides were assigned by SEQUEST HT (Eng et al., 1994), allowing a maximum of 2 missed tryptic cleavages, a minimum peptide length of 6, a precursor mass tolerance of 10 ppm, and fragment mass tolerances of .02 Da. Carbamidomethylation of cysteines and oxidation of methionine were specified as static and dynamic modifications, respectively. Protein detection required their identification in two technical replicates. Background proteins identified in Col‐0 plants were classified as contaminants and removed from the SUMO1/2 conjugate datasets.

## AUTHOR CONTRIBUTIONS

Theresé C. Rytz and Richard D. Vierstra designed the research. Theresé C. Rytz developed the transgenic material and performed the heat stress studies. Juanjuan Feng confirmed the genotypes and tested the sensitivity of the lines to various chemical. Jessica A.S. Barros conducted the MS/MS analyses. Richard. D. Vierstra and Theresé C. Rytz wrote the paper with input from the remaining authors. All authors edited the manuscript and approved the final version.

## CONFLICT OF INTEREST STATEMENT

The authors do not report any conflict of interest.

### PEER REVIEW

The peer review history for this article is available in the Supporting Information for this article.

## GRAPHS RENDERING

The graphs were generated in Microsoft Excel and presented in Adobe Illustrator.

## Supporting information


**Data S1.** Supporting information.

**Figure S1.** Location of the lysines in the 3D model of Arabidopsis SUMO1 that were replaced with arginines.
**Figure S2.** Arginine replacement of SUMO1 lysines does not impact the SDS‐PAGE profile of SUMO1/2 conjugates in Arabidopsis before and after heat shock.
**Figure S3.** Arginine replacement of SUMO1 lysines does not impact the sensitivity of root growth to hydroxyurea, mitomycin C, indole‐3‐acetic acid, or salicylic acid.
**Table S1.** Oligonucleotide primers used in this study.
**Table S2.** Full list of possible SUMOylated proteins affinity enriched from plants expressing 6His‐(M1R)‐SUMO1(H89R) and 6His‐(M1R)‐K0(H89R) and identified by MS/MS.

## Data Availability

The MS/MS dataset is available in Table S2.
