# Supplementary material for: Arabidopsis‐expressing lysine‐null SUMO1 reveals a non‐essential role for secondary SUMO modifications in plants
Source: Plant Direct. 2023 Jul 16;7(7):e506. doi: 10.1002/pld3.506 (PMC10350450; doi:10.1002/pld3.506)
Supplement: Supplementary file 2 — Figure S1. Location of the lysines in the 3D model of Arabidopsis SUMO1 that were replaced with arginines. Figure S2. Arginine replacement of SUMO1 lysines does not impact the SDS‐PAGE profile of SUMO1/2 conjugates in Arabidopsis before and after heat shock. Figure S3. Arginine replacement of SUMO1 lysines does not impact the sensitivity of root growth to hydroxyurea, mitomycin C, indole‐3‐acetic acid, or salicylic acid. Table S1. Oligonucleotide primers used in this study. Table S2. Full list of possible SUMOylated proteins affinity enriched from plants expressing 6His‐(M1R)‐SUMO1(H89R) and 6His‐(M1R)‐K0(H89R) and identified by MS/MS. [file PLD3-7-e506-s002.pdf]

SUPPLEMENTAL DATA

Arabidopsis expressing lysine-null SUMO1 reveals a non-essential role for secondary SUMO modifications in plants

Theresé C. Rytz, Juanjuan Feng, Jessica A.S. Barros, and Richard. D. Vierstra

Supplemental Table 1. Oligonucleotide Primers used in this Study

**Lysine→Arginine Codon Conversions**	
K9,10-R forward primer	CAAACCAGGAGGAAGACAGAAGGCCAGGAGAC
K9,10-R reverse primer	GTCTCCTGGCCTTCTGTCTTCCTCCTGGTTTG
K21,23-R forward primer	CAATCTCCGAGTCAGGGGACAGGTATCTC
K21,23-R reverse primer	GTCCC-CTGACTCGGAGATTGATGTGAGCTC
K35-R forward primer	GGTTTTCTTTAGGA-TCCGGAGAAGCACTCAGCTC
K35-R reverse primer	GAGCTGAGTGCTTCTC-CGGATCCTAAAGAAAACC
K41,42-R forward primer	GAAGCACTCAGCTCCGG-CGGCTGATGAATG
K41,42-R reverse primer	CATTCATCAGCCGCCGGAGCTGAGTGCTC
**K32,42-R mutagenesis**	
K23-R forward primer	CATCAATCTCAAAGTCCGGGGACAGGTATCTCTC
K23-R reverse primer	GAGAGATACCTGTCCCCGGACTTTGAGATTGAT
K42-R forward primer	GAAGCACTCAGCTCAAGCGGCTGATGAATGCTTAC
K42-R reverse primer	TAAGCATTCATCAGCCGCTTGAGCTGAGTGCTTC.
**PCR Detection of *WT* and *SUMO-K0* transgenes**	
P1 genomic primer	CGCCAAGCTATCAAACAAGTT
P2 genomic primer	GGAATTATCGAACCACTTTGTACAAG
P3 genomic primer	TTTCGTGTAGCTGCGATTAGG
P4 genomic primer	TTATCTTTGCTCGCCATTAGC
P5 genomic primer	GCCTTTTCAGAAATGGATAAATAGCCTTGCTTCC
P6 genomic primer	GTCGGAGAATCGGATTTCTTC
P7 genomic primer	TGAGGGTGTGTATTGGTGGAG
P8 genomic primer	ATTTTGCCGATTTCGGAAC
qRT-F *SUMO1* PCR Primer	CTGCAAACCAGGAGGAAGAC
qRT-R *SUMO1* PCR Primer	CATCGCATCGATCTCATCAC
qRT-F PCR *ACT2* Primer	GGCATCACACTTTCTACAATGAGC
qRT-R PCR *ACT2* Primer	ACCCTCGTAGATTGGCACAG

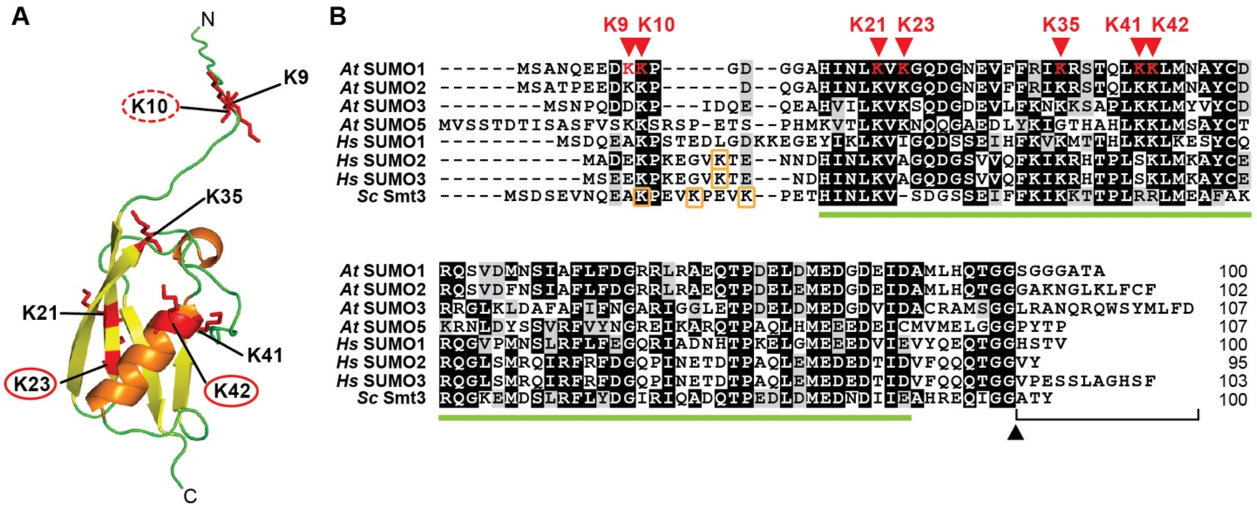

Supplemental Figure 1. Location of the lysines in the 3D model of Arabidopsis SUMO1 that were replaced with arginines.

(A) Predicted 3D structure of Arabidopsis SUMO1 shown in ribbon diagram generated with SWISS MODEL (http://swissmodel.expasy.org) using the model for human SUMO1 (PDB 2AWT) as the template. α-Helices, β-strands, and unstructured regions are colored in orange, yellow and green, respectively. The seven lysines are shown in stick form and colored in red. The circled residues are the lysines replaced in the K23,42-R variant. C, C-terminus. N, N-terminus. **(B)** Amino acid sequence alignment of the four expressed SUMO isoforms from *Arabidopsis thaliana* (*At*) along with orthologs from *Homo sapiens* (*Hs*) and *Saccharomyces cerevisiae* (*Sc*). Sequences were aligned with MAFFT version 7 (Katoh et al., 2019) and displayed in MacBoxShade (https://embnet.vital-it.ch/software/BOX_form.html. Identical and similar amino acids are shown in the black and grey boxes, respectively. Dashes denote gaps. The lysine$\rightarrow$arginine replacements are indicated by the red arrowheads. Additional N-terminal lysines shown to be SUMOylated in other organisms are highlighted by the orange boxes (Keiten-Schmitz et al., 2019; Vertegaal, 2010). The β-grasp Ub fold is located by the green line. The black arrowhead shows the diGly C-terminus of mature SUMOs after cleavage of the C-terminal extension (bracket). The numbers at the end indicate the residue lengths of the SUMO precursors before processing.

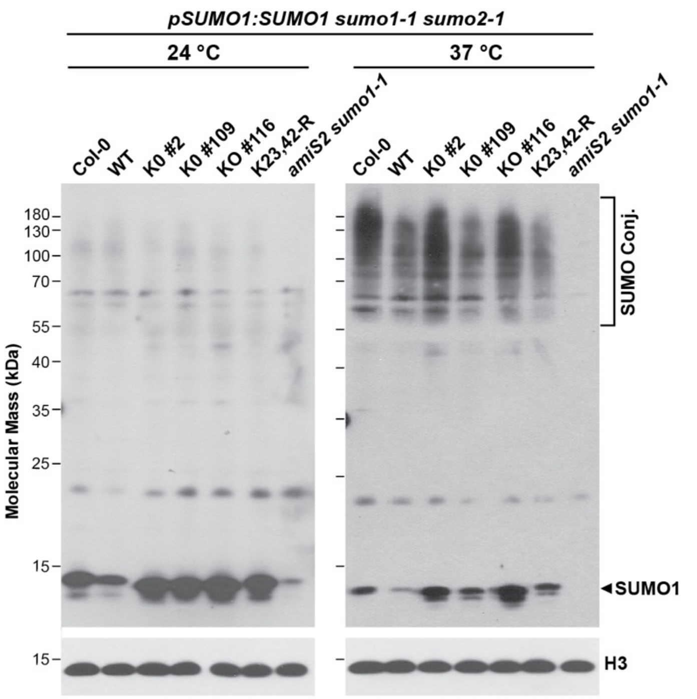

Supplemental Figure 2. Arginine replacement of SUMO1 lysines does not impact the SDS-PAGE profile of SUMO1/2 conjugates in Arabidopsis before and after heat shock.
Wild type (Col-0) seedlings and *sumo1-1 sumo2-1* seedlings expressing under the *SUMO1* promoter transgenes encoding wild-type SUMO1 (WT), the K23,42-R variant, or three independent lines expressing SUMO1-K0 (K0 32, K0 #109, and K0 #116), were grown for 7 days in liquid culture under continuous light at 24°C in 1/2 MS medium. At t = 0, the seedlings were either kept at 24°C (left panel) or subjected to a 30-min heat shock at 37°C followed by incubation at 24°C for 30 min (right panel). Response of the *amiS2 sumo1-1* line (van den Burg et al., 2010) was included for comparison. Free SUMO1 and SUMO1 conjugates were detected in total seedling extracts by immunoblotting with anti-SUMO1 antibodies (arrowhead and bracket, respectively). Exposure times in the left and right panels were adjusted to provide reasonably similar immunoblot signal intensities for free SUMO1. Immunodetection of histone H3 was used to verify near equal protein loading in each panel.

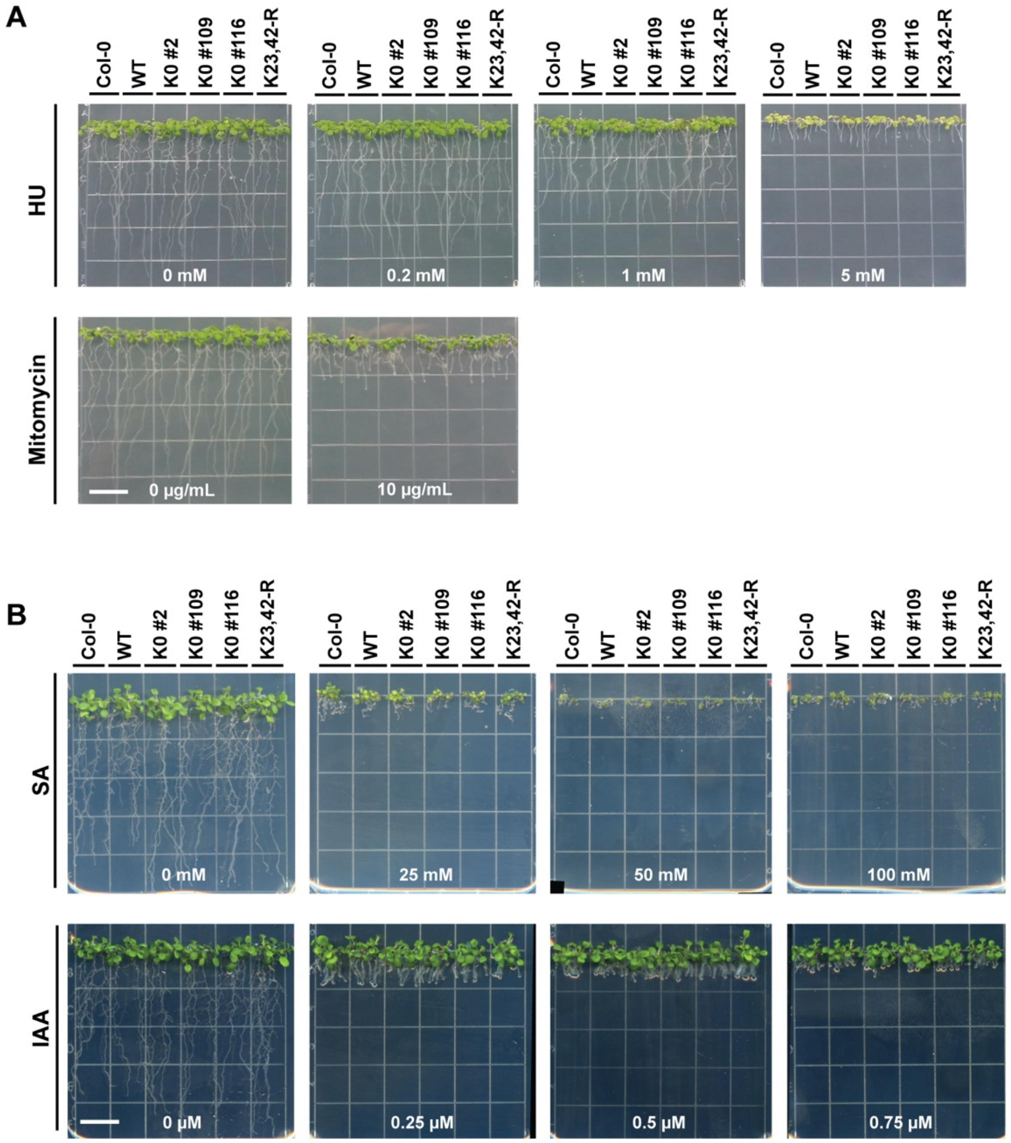

Supplemental Figure 3. Arginine replacement of SUMO1 lysines does not impact the sensitivity of root growth to hydroxyurea, mitomycin C, indole-3-acetic acid, or salicylic acid.

Wild type (Col-0) seedlings and *sumo1-1 sumo2-1* seedlings expressing under the *SUMO1* promoter transgenes either encoding wild-type SUMO1 (WT), the K23,42-R variant, or three independent lines expressing SUMO1-K0 (K0 32, K0 #109, and K0 #116), were grown for 10 days at 21°C under continuous light on solid GM medium containing 2% Suc, 0.8% agar, and the indicated concentrations of chemicals. Eight seeds from the indicated lines were planted in each sector. Scale bars = 1 cm.

(A) Hydroxyurea (HU) and mitomycin C.

(B) The auxin indole-3-acetic acid (IAA) and salicylic acid (SA).
